# Proteomic identification of early salicylate- and flg22-responsive redox-sensitive proteins in Arabidopsis

**DOI:** 10.1038/srep08625

**Published:** 2015-02-27

**Authors:** Pei Liu, Huoming Zhang, Boying Yu, Liming Xiong, Yiji Xia

**Affiliations:** 1 Department of Biology, Hong Kong Baptist University, Kowloon, Hong Kong; 2 Biological and Environmental Sciences and Engineering Division, King Abdullah University of Science and Technology, Thuwal, Saudi Arabia; 3 Biosciences Core Laboratory, King Abdullah University of Science and Technology, Thuwal, Saudi Arabia; 4 Partner State Key Laboratory of Agrobiotechnology, Chinese University of Hong Kong, Shatin, Hong Kong

## Abstract

Accumulation of reactive oxygen species (ROS) is one of the early defense responses against pathogen infection in plants. The mechanism about the initial and direct regulation of the defense signaling pathway by ROS remains elusive. Perturbation of cellular redox homeostasis by ROS is believed to alter functions of redox-sensitive proteins through their oxidative modifications. Here we report an OxiTRAQ-based proteomic study in identifying proteins whose cysteines underwent oxidative modifications in Arabidopsis cells during the early response to salicylate or flg22, two defense pathway elicitors that are known to disturb cellular redox homeostasis. Among the salicylate- and/or flg22-responsive redox-sensitive proteins are those involved in transcriptional regulation, chromatin remodeling, RNA processing, post-translational modifications, and nucleocytoplasmic shuttling. The identification of the salicylate-/flg22-responsive redox-sensitive proteins provides a foundation from which further study can be conducted toward understanding biological significance of their oxidative modifications during the plant defense response.

Upon recognition of pathogen infection, plant cells activate multifaceted defense responses. Invading pathogens are first perceived by host's pattern recognition receptors (PRRs) that detect pathogen-associated molecular patterns (PAMPs), leading to PAMP-triggered immunity (PTI)^1^. Flagellin from bacterial pathogens and chitin from fungal pathogens are among PAMPs that trigger PTI in plant cells. An early host response during PTI is increased production of reactive oxygen species (ROS) which occurs within several minutes of perception. Although this oxidative burst is weak and transient, it is believed to play an important role in activation of the immune response^2^,^3^. The mutation in FLS2, the PRR that recognizes flg22 (a peptide from flagellin), abolishes the flg22-induced oxidative burst and causes enhanced susceptibility to *Pseudomonas syringae*^3^. Pathogens have evolved various effector proteins that are delivered into hosts to suppress PTI, allowing the invading pathogens to successfully colonize hosts and cause disease. Many effectors have been found to suppress plant immunity by suppressing host cells' production of ROS^4^,^5^,^6^,^7^. For instance, the *Ustilago maydis* effector Pep1 suppresses the immune response by directly inhibiting host cell's peroxidase activity, thereby inhibiting ROS production^7^. Similarly, multiple HaRxLs effectors from *Hyaloperonospora arabidopsidis*, the effector AvrPiz-t from *Magnaporthe* oryzae, and the effector GrCEP12 from *Globodera rostochiensis* all suppress the flg22-induced generation of ROS^4^,^5^,^6^.

Plants have evolved hundreds of Resistance (R) proteins each of which can recognizes one or multiple corresponding effectors, leading to effector-trigger immunity (ETI)^1^. ETI leads to the hypersensitive response (HR) with strong activation of various defense mechanisms, often resulting in death of infected cells, a phenomenon termed the hypersensitive cell death. A sustained high level accumulation of ROS is associated with ETI and occurs around 2 hours following infection by avirulent pathogens that trigger ETI. ROS, together with nitric oxide, is essential for the hypersensitive cell death and activation of many defense-related genes and other defense mechanisms^8^,^9^. ROS production during PTI and ETI is mediated by multiple ROS generating systems including NADPH oxidases and apoplastic peroxidases^10^.

A number of defense signaling molecules are accumulated during the HR, among which salicylate and jasmonate are best known. Salicylate and jasmonate are considered plant defense hormones and, together with almost all other phytohormones, modulate the defense pathways in plants with outcomes dependent on different types of pathogens and the genetic constitution of the host^11^. Salicylate activates the defense response at the infected site and systemic acquired resistance^12^. Salicylate treatment potentiates the oxidative burst and causes perturbation of the cellular redox states^13^,^14^. NONEXPRESSER OF PR GENES 1 (NPR1), a key player in plant defense, alters its redox state in response to endogenous salicylate accumulation or exogenous salicylate treatment and translocates from the cytosol to nucleus to activate transcription of defense genes^15^.

Despite well recognized importance of ROS in the plant defense responses, the precise roles of ROS and the defense pathways they modulate remain elusive. ROS likely play their roles through oxidative modifications of proteins that are sensitive to perturbation of cellular redox states. Identification of redox-sensitive proteins would be an important step toward understanding the roles of redox regulation and redox signaling in the plant defense responses.

Proteomics approaches have become available in the last decade for identification of proteins that undergo oxidative modifications in cells subjected to various redox perturbation treatments^16^. A commonly used strategy in redox proteomic methods, whether it is gel-based or gel-free, is to first block free thiol groups with alkylating reagents such as N-ethylmaleimide (NEM) or iodoacetamide (IAM) during protein extraction, followed by reduction of reversibly oxidized cysteine residues which are then labeled with differential alkylating reagents for detection and identification. Gel-free methods such as OxICAT^17^, cysTMT^18^, cysTRAQ^19^, OxiTRAQ^20^ and OxMRM^21^, which offer higher sensitivity and resolution, have increasingly been used for identification and quantification of redox-sensitive proteins.

We previously developed gel-based and the OxiTRAQ approaches for identification of proteins that underwent oxidative modifications in Arabidopsis cells subjected to treatment with hydrogen peroxide^20^,^22^. Here, we report our study on the identification of proteins in Arabidopsis cells whose cysteines underwent reversible oxidative modifications in an early response to the treatment with salicylate and flg22.

## Results and discussion

### The identification of the salicylate- and/or flg22-responsive redox-sensitive proteins

Redox proteomics approaches are generally based on differential labeling of reduced and oxidized cysteines with thiol-reactive reagents. We attempted to identify proteins whose cysteines undergo reversible oxidative modifications in Arabidopsis cells subjected to the treatment with salicylate or flg22 using a redox proteomics approach termed OxiTRAQ that was developed in our previous study^20^. Arabidopsis suspension cells were treated with 10 μM flg22 or 0.5 mM salicylate for a short period (15 min) and then harvested for protein extraction and processing. The short treatment was intended for identifying early-responsive proteins as well as for minimizing the chance in a change in protein levels that might complicate determination of their changes in redox states. Proteins were extracted in presence of N-ethylmaleimide to block reduced thiols to prevent their oxidation during extraction. Reversibly oxidized thiols in the protein samples were then reduced by DTT and labeled by a biotinylated thiol-reactive reagent. Biotin-tagged cysteine-containing peptides were affinity-purified and labeled with iTRAQ reagents for mass spectrometry identification and quantification. Three biological replicates were included in this study, and each biological replicate was analyzed three times (as three technical replicates) using a paralleled HCD-CID fragmentation mode in a LTQ-Orbitrap. A higher abundance for a particular peptide indicates that a higher portion of it was in a reversibly oxidized state in the sample which was tagged by biotin following their reduction by DTT. Therefore, a difference in abundance of a cysteine-containing peptide between salicylate- or flg22-treated cells and the mock-treated control cells represents the difference of its redox states in those cells. This approach only quantifies reversibly oxidized thiols that are DTT-reducible.

Through the OxiTRAQ analysis, we identified a total of 7315 unique cysteine-containing peptides which are from 2986 distinct proteins in at least two biological replicates with a false discovery rate of less than 1% at both peptide and protein levels (Supplementary Table S1). A peptide whose abundance differed by more than 1.5-fold with a *p* value of <0.05 (two tailed student's t-test) was considered to be redox-sensitive in this report. With these criteria, 128 cysteine-containing peptides from 111 different proteins were found redox-sensitive in response to the salicylate or flg22 treatment (Supplementary Table S2). Among those 128 putative redox-regulated cysteine-containing peptides that are listed in Table Supplementary S2, 106 peptides from 97 proteins and 56 peptides from 49 proteins were identified as redox-sensitive to the treatment of salicylate and flg22, respectively. More than one cysteine-containing peptide was identified in some proteins. The results suggest that the 0.5 mM salicylate treatment caused more pronounced perturbation of cellular redox homeostasis than the 10 μM flg22 treatment. Besides, 33 of the redox-sensitive peptides were identified in both treatments, suggesting that there are shared and distinct responses to these two treatments. Three of these redox-sensitive proteins were in more reduced states under the treatment of either salicylate or flg22 whereas the others were in more oxidized states, indicating that the salicylate and flg22 treatments largely made the cells in a more oxidized status. The identification of the proteins in a more reduced state under the treatments might also suggest a dynamic nature of protein redox states.

Intra-molecular disulfide bonds of identified proteins were predicted by DiNNA (http://clavius.bc.edu/~clotelab/DiANNA/). Fifty one of the 128 redox-sensitive peptides were predicted to form intra-molecular disulfide bonds. Besides forming disulfide bonds, it should be noted that other modifications, such as glutathionylation, nitrosylation, and oxidation to sulfenic acid, are also chemically reversible by DTT which was used to reduce oxidized thiols before their tagging by the biotinylated reagent. Some of these reversible thiol modifications identified through this proteomics method could be due to any of those types of modifications.

### Quantification of protein levels in response to the salicylate and flg22 treatment

**Figure 1:**
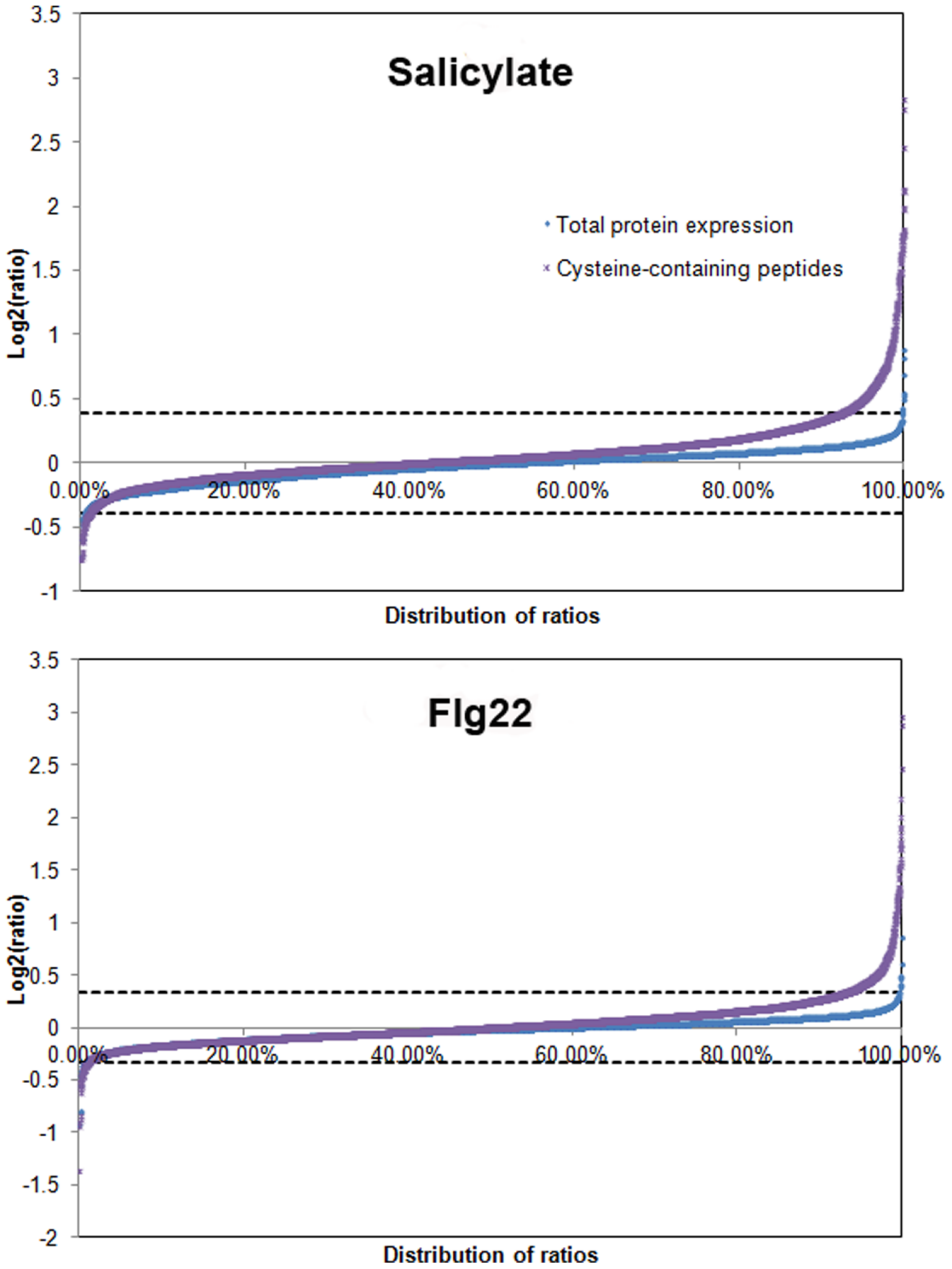
Ratio distribution of OxiTRAQ and iTRAQ data. The average ratios for cysteine-containing peptides and total proteins were log2-transformed and plotted to equally spaced bins based on the ratio values. The top graph shows ratio distribution of cysteine-containing peptides and total proteins of the flg22-treated samples and the bottom graph shows those of the salicylate-treated samples.

Since the OxiTRAQ method is based on the difference in the abundance of a peptide that is reversibly oxidized in the different samples and biotin-tagged during the protein processing, a difference in global protein abundance could lead to a false positive or false negative result. Therefore, a regular proteomic experiment using the iTRAQ analysis was conducted in parallel to monitor abundance of proteins. The analysis included two biological replicates, and each biological replicate was analyzed by mass spectrometry three times (as technical replicates). A total of 4525 and 4497 proteins were identified from the two biological replicates, respectively, each of which included a control, a salicylate-treated sample, and a flg22-treated sample. Among them, 3573 proteins were shared in both biological replicates (Supplementary Table S3). The average ratios from the OxiTRAQ data and the regular iTRAQ data were log2-transformed and plotted to equally spaced bins based on the ratio values (Figure 1). More than 99% of regular iTRAQ ratios (99.3% for salicylate and 99.2% for flg22) remained within 3 standard deviation of the mean (i.e. ratio <0.76 or >1.31 for salicylate and ratio <0.79 or >1.27 for flg22). The results indicated that only a few proteins changed abundance after the 15 minute salicylate or flg22 treatment. These data distributions, together with the comparison between the abundance of peptides from the OxiTRAQ analysis and that of the corresponding proteins from the iTRAQ data, indicated that relative abundance of each cysteine-containing peptide represents its redox state with a few exceptions (see below). Compared to 98% cysteine-containing peptides out of all the identified peptides (6539 out of 6610) in one replicate of OxiTRAQ, there are about 15% cysteine-containing peptides in the global iTRAQ analysis (2790 out of 18596). Therefore, more cysteine-containing peptides were identified through the OxiTRAQ method.

We detected a total of eight proteins that showed a significant change in the protein levels following either salicylate or flg22 treatment (Supplementary Table S4), indicating that the short time treatments had yet to cause a major change in protein synthesis or degradation. Among the eight proteins, the level of AtPIN7 (an auxin efflux carrier protein) was decreased in both treatments. The change in the AtPIN7 levels following the salicylate and flg22 treatment suggests involvement of auxin movement in the early defense response. The role of auxin in disease resistance is complex; however, auxin signaling and transport is generally considered antagonistic with the role of salicylate (for reviews, see Refs. 11,23). The other six proteins that showed a change in abundance include a global transcription factor group E4, a zinc finger (C3HC4-type RING finger) family protein, transmembrane proteins 14C, a PDI-like protein, and two proteins involved in vesicle trafficking: an exocyst complex family protein and endoplasmatic reticulum retrieval protein 1B. Vesicle trafficking has increasingly been recognized to be an important mechanism in the plant defense pathways and pathogenesis, including in the flagellin-FLS2-mediated immunity (for a review, see Ref. 24). The abundance of the cysteine-containing peptides of these eight proteins from the OxiTRAQ analysis was then normalized based on the expression levels of the corresponding proteins.

### Categorization of the redox-sensitive proteins identified from the OxiTRAQ analysis

Among the 111 salicylate- or flg22-responsive redox-sensitive proteins, at least 28 proteins have previously been reported to undergo oxidation under oxidative stress treatments (Supplementary Table S2). Among three of the 108 proteins that were in a more reduced state are expansin B1 and reversibly glycosylated polypeptide 1 involved in cell structure control, and pyruvate decarboxylase 2 involved in alcohol fermentation. The other 105 proteins that were in more oxidized states are classified into various functional categories and discussed below. Table 1 contains a list of the redox-sensitive peptides with a higher than 2.5 fold change.

### Transcriptional and post-transcriptional regulation

Among the salicylate- and/or flg22-responsive redox-sensitive proteins, 9 proteins are involved in transcriptional and post-transcriptional regulation. These include three transcription factors, two histone deacetylases (HDA19 and HDA9), one splicing factor Prp18 family protein, and several other RNA-processing proteins. General regulatory factor 6 (GRF6) and GRF8 were previously reported to be Trx- and Grx-targeted proteins, respectively^25^,^26^. Our study showed that the cysteine residues in a conserved region of GRF3 (IC*DGILNVLEAHLIPSASPAESK) became oxidized in the salicylate-treated cells. It is plausible that the cysteines in this conserved region in other GRFs could also be sensitive in response to similar oxidative stresses.

Chromosome remodeling and epigenetic control are important mechanisms in plant response to biotic and abiotic stresses^27^,^28^. Histone acetylation has a key role in chromosome remodeling. In this study, HDA19 and HDA9 were found to be oxidized upon the salicylate treatment. The oxidation of HDA19 and HDA9 could render them inactive, thereby elevating histone acetylation and expression of associated genes. Although histone acetylation mostly promotes gene expression, its role in plant defense is complicated. For instance, histone deacetylation by Arabidopsis HDA19, which interacts with several transcriptional (co)regulators in plant defense, represses expression of some defense-related genes but promotes expression of others^29^,^30^,^31^. The outcome might be dependent on whether the defense regulatory genes directly controlled by a HDA are activators or repressor of the defense pathway.

Alternative mRNA splicing can result in a diversity of functional proteins by a single gene. Stress responsive genes are particularly prone to be alternatively spliced in plants^32^. For instance, alternative splicing of Toll-like resistance genes play a crucial role in immunity pathways^33^. A splicing factor Prp18 family protein showed oxidative modification under salicylate treatment. It will be interesting to know whether oxidation of the splicing-related protein alters splicing patterns of their target genes.

### Translational and post-translational processes

Protein synthesis is one of the most energy-consuming cellular processes and is an important regulation step in stress responses. Several proteins involved in protein synthesis have been found to undergo oxidative modifications in response to treatment of hydrogen peroxide^20^,^22^. Two elongation factors were found to be oxidized in the salicylate-treated cells. One of them (GTP binding elongation factor Tu family protein) was previously found to also undergo oxidation in the H_2_O_2_-treated Arabidopsis cells^22^ and is encoded by four Arabidopsis genes that code four proteins with the identical amino acid sequences. Several ribosomal proteins and aminoacyl-tRNA ligases were oxidized in response to the salicylate- and/or flg22- treatment.

In addition to alternative splicing, post-translational modification (PTM) of proteins is another way to increase the diversity of proteins. Some protein phosphatases are regulated by regulatory subunits, which cause allosteric conformational changes of the phosphatase, thereby switching between their active and inactive states^34^. Three regulatory subunits of protein phosphatases were oxidatively modified under the salicylate treatment, including protein phosphatase 2A subunits A1/RCN1, A2, and A3. The identified redox-senstive cysteines are in a conserved site in these three proteins, suggesting that they are similarly regulated through oxidative modifications. Many studies have shown that Ser/Thr phosphatase type 2A participates in biotic and abiotic stress response in plant^35^. It is possible that the oxidized state of the regulatory subunits might render them functionally inactive, which might enhance phosphorylation of targeted proteins.

More than one dozen of the redox-sensitive proteins are predicted to be involved in protein folding and proteolytic processing, which include several proteases/peptidases, heat shock proteins (HSP)/chaperones, and protein disulfide isomerase (PDI). HSPs and PDIs function in protein folding within cytoplasm and the endoplasm reticulum, respectively. Hsp33 is known to be redox-regulated and forms a dimer upon exposure to oxidants which then binds to substrate proteins to prevent their aggregation^36^. In this study, a chaperonin-60 protein TCP1 and a HSP81 protein were found to be oxidized under salicylate or flg22 treatment. PDI proteins, which are ubiquitous thiol-disulfide oxidoreductases in all eukaryotic cells, catalyze the formation, reduction and isomerization of disulfide bonds in proteins. The classical PDI consists of two conserved active sites (CGHC), one near their N-termini and the other near their C-termini^37^. The cysteines in both conserved sites of AtPDIL1-1 and AtPDIL2-1 were identified as redox-sensitive. These PDIs were previously found to be oxidized in the H_2_O_2_-treated Arabidopsis cells^22^.

### Antioxidant systems and pathogen defense

Among the redox-sensitive proteins related to the antioxidant system are thioredoxin superfamily proteins, peroxiredoxins (Prxs), and a peroxidase superfamily protein. Some of these proteins are involved in scavenging ROS or reduction of other proteins. They might become oxidized while acting to reduce oxidized proteins or other biomolecules caused by the salicylate- or flg22-treatment. Two peroxiredoxins, peroxiredoxin IIF (AtPrxIIF) and 2-cysteine peroxiredoxin B (2-Cys Prx B), were identified in this study. Prxs use a conserved peroxidatic cysteine residue (C_P_) to reduce peroxide substrates resulting in the oxidation of C_P_, while a resolving Cys (C_R_) then forms a disulfide bond with C_P_ to regenerate reduced C_P_^38^. The cysteine we identified in 2-Cys Prx B is the C_R_ residue located at the C-terminal region. AtPrxIIF, a Prx that is targeted to plant mitochondria^39^, is an important H_2_O_2_-scavenging enzyme. The cysteine residue (Cys-64) that became oxidized in response to the salicylate and flg22 treatments belongs to one of two conserved catalytic cysteines for substrate-dependent thiol-disulfide transition which can be regenerated by glutathione^40^.

Several important regulatory proteins in the defense pathways, such as NPR1, TGA1, and TGA2, are known to be redox-regulated^15^,^41^ but were not identified in this study as redox-sensitive proteins. One possibility is that the OxiTRAQ method is not sensitive enough to detect changes in redox states of very low abundant proteins. Another possibility is that they did not undergo oxidative modifications under the short period of the SA or flg22 treatment.

Interestingly, LysM domain-containing GPI-anchored protein 2 (LYM2) was found to be oxidized in response to both salicylate and flg22 treatments. LYM2 is the Arabidopsis homolog of a rice chitin receptor-like protein; however whether it functions in chitin reception in Arabidopsis remains elusive although it is known to function in defense pathways, particularly in defense against fungal pathogens^42^,^43^. It was recently shown that LYM2 functions in defense responses by regulating chitin-triggered molecular flux between cells^44^. The oxidative modification of LYM2 under the salicylate and flg22 treatment suggests that LYM2 might also participate in the flagellin-mediated immunity although the biological consequence of its oxidation remains unknown.

Four cysteine residues in non-specific LTP1 (lipid transfer protein 1) became oxidized in response to flg22. Plant LTPs are small cysteine-rich proteins and often grouped into the PR14 family due to their role in plant defense^45^. They possess eight conserved cysteine residues to form four disulfide bridges which stabilize the three-dimensional structure^46^.

### Cell structure, transport, and vesicle trafficking

Several redox-sensitive proteins are involved in cellular structure, including two actin depolymerizing factors (ADF1 and ADF4), tubulin α, annexin, expansin, and a cellulase. ADF3, annexin and tubulin α have been reported to undergo S-nitrosylation^47^. ADF4, a regulator of actin cytoskeletal dynamics, is known to be required for activation of R gene-mediated resistance in Arabidopsis^48^. ADFs have been found to be localized in both the cytoplasm and the nucleus^49^, and their translocation into nucleus is facilitated by cytochalasin D^50^. It would be interesting to know whether their reduction/oxidation might mediate their nucleocytoplamic shuttling. Annexins are Ca^2+^-dependent membrane-binding proteins and annexins 1 was previously found to be susceptible to oxidant-driven *S*-glutathionylation^51^,^52^. Abscisic acid treatment also led to its S-glutathionylation^51^.

Nucleocytoplamic shuttling is an important mechanism in transducing stress stimuli into transcriptional reprogramming as well as for transporting RNA for protein synthesis. These processes are mediated through nuclear pore complexes composed of nucleoporin proteins (NUPs)^53^,^54^. We identified three redox-sensitive proteins that are involved in nucleocytoplasmic trafficking, including RAS-related nuclear protein-1, suppressor of auxin resistance 1 (also known as NUP160), and ATKPNB1. AtKPNB1 is a homolog of human KPNB1 (importin β1) that interacts with importin α proteins, nucleoporin AtNUP62 and Ran proteins to mediate transportation of cargoes into the nucleus and expression of stress-induced genes in response to environmental stimuli^55^. NUP160 is needed for nuclear mRNA export and is also an important component of the EDS1-dependent defense pathway in Arabidopsis^54^.

### Metabolism and energy production

A larger number of the redox-sensitive proteins fall into this category. Many of these proteins have previously been reported to undergo oxidative modifications, including enolase^22^, fructose-bisphosphate aldolase 1^56^, and purine biosynthesis 4^20^. Two cysteines of CP12 were in a more oxidized state under salicylate treatment. CP12 is a small chloroplast redox-sensitive protein that interacts with glyceraldehyde-3-phosphate dehydrogenase (GAPDH) and phosphoribulokinase (PRK) to form a complex and their association and dissociation is regulated by the redox state of Trx via oxidation and reduction of two cysteine pairs on the CP12 protein^57^. Oxidation of CP12 could induce formation of the complex and reduce the activity of both PRK and GAPDH. In this study, we used suspension cells growing under dark for the analysis. The biological consequence of the oxidation of CP12 under the elicitor treatment that might affect the Calvin cycle is unknown. We previously identified two GAPDH C subunits (GAPC-1 and GAPC-2) as profoundly oxidized proteins in Arabidopsis cells under H_2_O_2_ treatment^22^. However, we did not detect its oxidation in this study. One possibility is that the oxidation of GAPDH, even if it occurred under the salicylate and flg22 treatment, could be irreversible as its H_2_O_2_-triggered oxidization was apparently irreversible^22^. Other redox sensitive proteins identified in this study include three S-adenosyl-L-methionine-dependent methyltransferases superfamily proteins and a xanthoxin dehydrogenase (ABA2). ABA2, an abscisic acid (ABA) biosynthetic enzyme that converts xanthoxin to ABA-aldehyde, was found to be in a more oxidized state in response to both salicylate and flg22 treatments, suggesting that redox regulation of ABA biosynthesis might play a role in the defense response.

### Concluding remarks

Treatment of Arabidopsis cells with two inducers of plant immunity, salicylate and flg22 that are known to affect cellular redox homeostasis, led to oxidative modifications of a variety of proteins. Some of these redox-sensitive proteins were identified from one of these two treatments whereas others were found oxidized in response to both treatments, suggesting overlapping and distinct pathways in the salicylate- and flg22-mediated redox control. The salicylate treatment (0.5 mM) caused more pronounced perturbation of cellular redox homeostasis than the fl22 treatment (10 μM) since a larger number of oxidized proteins were identified from the salicylate-treated cells. Among the redox-sensitive proteins identified in the study include many regulatory proteins such as chromatin remodeling proteins, transcription factors, mRNA processing proteins, and those involved in post-translational protein modifications. The identification of these proteins and further understanding of the biological consequences of their oxidative modifications would greatly contribute to our understanding of the role of redox signaling in the immune response in plants.

## Methods

### Cell culture and stress treatments

Suspension cells of *Arabidopsis thaliana* (Columbia ecotype) T87 was obtained from Liwen Jiang laboratory (Chinese University of Hong Kong, HK). The cells were maintained as previously described^20^. Experiments were performed 4 days after subculturing. Salicylate was added to the cell culture at a final concentration of 0.5 mM. Treatment with flg22 was performed in the same way, except at a final concentration of 10 μM. For the control, the same amount of water was added. After 15 min incubation, the stress-treated and control cells were harvested by filtration, washed with the wash buffer [20 mM Tris-HCl (pH = 8.0), 5 mM EDTA, 0.5% Triton X-100, 100 mM NaCl], and immediately frozen in liquid nitrogen for protein extraction. Three biological replicates were included for each treatment.

### Protein extraction

Protein extraction for the OxiTRAQ study was performed according to the procedure described before^20^. Briefly, approximately 300 mg cells were ground in liquid nitrogen and incubated with 1 mL extraction buffer [20 mM Tris-HCl (pH 7.0), 5 mM EDTA, 0.5% Triton X-100, 100 mM NaCl, 1% SDS, and 1% protease inhibitor cocktail (Sigma-Aldrich, St Louis, MO, USA)] supplemented with 10 mM N-ethylmaleimide (NEM). Proteins were precipitated by addition of 5 volumes of iced acetone. After centrifugation, the supernatant was discarded. Protein pellets were re-suspended in the protein extraction buffer and quantified using DC Protein Assay Kit II (Bio-Rad, Hercules, CA, USA). For the iTRAQ analysis of total protein abundance, the procedure for protein extraction was the same, except that there was no NEM in the extraction buffer.

### Biotin tagging, purification, and iTRAQ labeling for OxiTRAQ assay

Approximately 3 mg proteins of each sample were reduced with 10 mM dithiothreitol (DTT) at 37°C for 30 min. DTT was then removed by Zeba Spin desalting columns (Thermo Scientific, Rockford, IL, USA). The reduced protein sample was alkylated with 0.4 mM of thiol-reactive N-[6-(Biotinamido) hexyl]-3'-(2'-pyridyldithio) propionamide (biotin-HPDP) (Thermo Scientific) for 1 hour at room temperature (RT) in the dark. Proteins were then acetone-precipitated and re-solubilized in 8 M urea. The sample was diluted eight times with 50 mM triethylammonium bicarbonate (TEAB) and digested with trypsin (Sigma) for overnight at 37°C. Digestion was terminated by 1% trifluoroacetic acid (TFA), desalted using Sep-Pak C18 cartridges (Waters, Milford, MA, USA) and dried in a SpeedVac (Eppendorf, Hamburg, Germany). The resulting peptides were dissolved in incubation buffer [20 mM Tris-HCl (pH = 7.7), 1 mM EDTA, 0.4% Triton X-100, 100 mM NaCl] and incubated with 250 μL NeutrAvidin agarose resin (Thermo Scientific) at RT for 1 hour. The resin was washed 3 times with 1 mL wash buffer (the incubation buffer containing 600 mM NaCl), followed by wash with 5 mM NH_4_HCO_3_/20% acetonitrile for 2 times. The peptides were eluted with 800 μL of 10 mM DTT. The eluted sample was alkylated with 55 mM iodoacetamide (IAM), desalted using Sep-Pak C18 cartridges, and dried in a SpeedVac. The isolated peptides were labeled with iTRAQ reagents according to the manufacturer's instructions (Applied Biosystems, Framing-ham, MA, USA). The control, salicylate- and flg22-treated samples were labeled with iTRAQ reporter reagents 114, 115 and 116, respectively. The labeled mixtures were fractionated by strong cation exchange as previously described^58^. Three biological replicates were included for each treatment.

### iTRAQ for total protein abundance assay

For determining abundance of proteins, 200 μg proteins from each sample was reduced by 10 mM DTT at 37°C for 30 min and alkylated by 55 mM IAM at RT in the dark for 30 min. The samples were diluted with 50 mM TEAB, followed by digestion with trypsin at a 1:40 trypsin-to-protein mass-ratio at 37°C for 12–16 hours. After digestion was stopped by 1% TFA, peptides were desalted by Sep-Pak C18 cartridges, and dried in a SpeedVac. The peptides were then labeled with iTRAQ reagents and fractioned by strong cation exchange as described above. Two biological replicates were included for each treatment.

### LC-MS/MS analysis and data analysis

Each sample was analyzed three times as technical replicates by an Easy-nLC (Thermo Scientific) coupled to an LTQ-Orbitrap Velos (Thermo Scientific) and the MS data were searched using Mascot (version 2.3) as described before^20^. A peptide with a Mascot expectation value of less than 0.05 was used for further quantification analysis. Normalization based on summed intensities was performed to make sure the total intensities of each of the iTRAQ channels were equal. The spectral intensity of a corresponding cysteine-containing peptide from three technical replicates of one biological replicate was combined and summed, and the peptide ratio was calculated from the summed values. Proteins identified and quantified at least in two biological repeats were used for further analysis. Student's t-test (two tails) was performed on the log2-transformed peptide ratio for statistical analysis. A peptide/protein with a ratio more than 1.5-fold and a *p* value of <0.05 was considered significantly changed. The mass spectrometry proteomics data have been deposited to the ProteomeXchange Consortium^59^ via the PRIDE partner repository with the dataset identifier PXD001349.

## Author Contributions

P.L. and H.Z. performed all the experiments including sample preparation, mass spectrometry analysis, and data analysis. B.Y. was involved in sample preparation. Y.X. and H.Z. outlined the research plan, and the study was under supervision of Y.X. and L.X., P.L. and H.Z. developed the main body of the manuscript and all authors participated in revising the manuscript.

## Supplementary Material

Supplementary Information Supplementary Table 1

Supplementary Information Supplementary Table 2

Supplementary Information Supplementary Table 3

Supplementary Information Supplementary Table 4

## Figures and Tables

**Table 1:** List of a subset of the salicylate- and/or flg22-responsive redox-sensitive peptides. The cysteine carbamidomethylation are marked with an asterisk symbol (*), cysteine N-ethylmaleimide with a hash symbol (#) in the peptide sequence and the ratios that are statistically significant are marked as bold

Protein ID	Protein description	Peptide sequence	SA	flg22
Transcriptional and post-transcriptional regulation				
AT4G38130.1	HDA19| histone deacetylase 19	LNHGLC*DIAINWAGGLHHAK	**2.70**	1.86
AT1G58050.1	RNA helicase family protein	LLFC*TTGILLR	**2.49**	**2.54**
AT1G68290.1	ENDO 2 | endonuclease 2	EGHEIIC*K	**2.73**	0.96
AT1G68290.1	ENDO 2 | endonuclease 2	WETC*TKK	**2.55**	0.81
Translation				
AT5G10360.1	RPS6B | Ribosomal protein S6e	GC*IVSPDLSVLNLVIVKK	**3.01**	2.44
AT5G10360.1	RPS6B | Ribosomal protein S6e	GC*IVSPDLSVLNLVIVK	**3.13**	**3.45**
AT3G04840.1	Ribosomal protein S3Ae	NVLC*QFWGMDFTTDKLR	**2.80**	1.66
AT1G09620.1	aminoacyl-tRNA ligases	LIVPIC*PHFADYVWR	**2.70**	**2.85**
Posttranslational modifications and protein folding				
AT1G21750.1	ATPDIL1-1| PDI-like 1-1	NVLLEFYAPWC*GHC*QK	**2.68**	1.89
AT1G63770.2	Peptidase M1 family protein	VYSLIGGFC*GSPVNFHAK	**3.04**	2.21
AT5G20660.1	Zn-dependent exopeptidases superfamily protein	VLERLPPFC*TMFGK	3.08	**3.34**
AT5G17780.1	alpha/beta-Hydrolases superfamily protein	C*VC*FIIC*K	**2.60**	0.89
Antioxidant systems and pathogen defense				
AT1G65980.1	TPX1 | thioredoxin-dependent peroxidase 1	KVILFGVPGAFTPTC*SMK	**3.32**	2.47
AT1G70580.1	AOAT2| alanine-2-oxoglutarate aminotransferase 2	GVMQILNC*VIR	**3.41**	2.16
AT1G23310.1	AOAT1| glutamate:glyoxylate aminotransferase	GVMQILNC*VIR	**3.47**	2.23
Metabolism				
AT2G47400.1	CP12-1| CP12 domain-containing protein 1	ETC*ADDPVSGEC*VAAWDEVEELSAAASHAR	**2.95**	1.22
AT2G36530.1	LOS2, ENO2 | Enolase	LGANAILAVSLAVC*K	**2.70**	1.89
AT2G19940.1	oxidoreductases	LVANPGC*YPTTIQLPLVPLLK	**3.99**	2.05
AT2G39270.1	probable adenylate kinase	NFHWVFLGC*PGVGK	**2.80**	2.07
AT5G49810.1	MMT| methionine S-methyltransferase	FC*SLIAGFMR	**3.50**	2.68
AT3G63410.1	APG1|MPBQ/MSBQ methyltransferase	AC*LIGPVYPTFWLSR	**5.54**	**5.56**
AT1G31850.1	putative methyltransferase PMT20	SDYNKLQSLLTSMC*FK	**3.20**	2.74
AT3G42050.1	vacuolar ATP synthase subunit H family protein	GVPIAISC*LSSLLKEPVVR	**7.15**	**7.81**
Cell structure				
AT3G46010.1	ADF1| actin depolymerizing factor 1	IFFIAWC*PDIAK	**3.94**	2.27
AT2G46930.1	Pectinacetylesterase family protein	WLIQLEGGGWC*NTR	**3.52**	**1.8**
Cellular transport and signal transduction				
AT5G20010.1	RAN-1| RAS-related nuclear protein-1	KYEPTIGVEVHPLDFFTNC*GK	**2.69**	1.76
AT3G43300.1	ATMIN7| HOPM interactor 7	SLIVDC*IVQMIK	**3.32**	1.8
Others				
AT2G21620.1	RD2 |dessication responsive protein	HAFDWALVHFC*R	**2.99**	2.35
AT5G11880.1	Pyridoxal-dependent decarboxylase family protein	SLIANTC*C#FVNHVTGVK	**2.66**	1.9
AT2G41800.1	unknown	LHDFGHLC*GPVLDSVVVTLAR	**4.38**	2.31
